# Supplementary material for: Chemical Profile and Promising Applications of Cucurbita pepo L. Flowers
Source: Antioxidants (Basel). 2024 Nov 30;13(12):1476. doi: 10.3390/antiox13121476 (PMC11673392; doi:10.3390/antiox13121476)
Supplement: Supplementary file 1 [file antioxidants-13-01476-s001.zip › antioxidants-3214671-supplementary.pdf]

Supplementary materials:

Table S1. Correlation between TPC and outcomes obtained by the DPPH, FRAP and ABTS methods. The correlation coefficients were evaluated by using Pearson's method.

Antioxidant Assay	TPC
	R^2
DPPH	0.98
ABTS	0.98
FRAP	0.98
